# Genomic and Evolutionary Analysis of *Salmonella enterica* Serovar Kentucky Sequence Type 198 Isolated From Livestock In East Africa

**DOI:** 10.3389/fcimb.2022.772829

**Published:** 2022-06-20

**Authors:** Mauro de Mesquita Sousa Saraiva, Valdinete Pereira Benevides, Núbia Michelle Vieira da Silva, Alessandro de Mello Varani, Oliveiro Caetano de Freitas Neto, Ângelo Berchieri, Enrique Jesús Delgado-Suárez, Alan Douglas de Lima Rocha, Tadesse Eguale, Janet Agnes Munyalo, Samuel Kariuki, Wondwossen Abebe Gebreyes, Celso José Bruno de Oliveira

**Affiliations:** ^1^ Department of Pathology, Theriogenology, and One Health, Sao Paulo State University (FCAV-Unesp), Jaboticabal, Brazil; ^2^ Department of Preventive Veterinary Medicine, College of Veterinary Medicine, The Ohio State University, Columbus, OH, United States; ^3^ Department of Animal Science, Center for Agricultural Sciences, Federal University of Paraiba (CCA/UFPB), Areia, Brazil; ^4^ Department of Technology, Sao Paulo State University (FCAV-Unesp), Jaboticabal, Brazil; ^5^ Department of Preventive Veterinary Medicine, Veterinary School, Federal University of Minas Gerais (UFMG), Belo Horizonte, Brazil; ^6^ Facultad de Medicina Veterinaria y Zootecnia, Universidad Nacional Autónoma de México (UNAM), Mexico City, Mexico; ^7^ Aklilu Lemma Institute of Pathobiology, Addis Ababa University, Addis Ababa, Ethiopia; ^8^ Centre for Microbiology Research, Kenya Medical Research Institute, Nairobi, Kenya; ^9^ Global One Health Initiative (GOHi), The Ohio State University, Columbus, OH, United States

**Keywords:** antimicrobial resistance, foodborne pathogen, genetic diversity, *Salmonella* pathogenicity islands, salmonellosis, whole genome sequencing

## Abstract

Since its emergence in the beginning of the 90’s, multidrug-resistant (MDR) *Salmonella enterica* subsp*. enterica* serovar Kentucky has become a significant public health problem, especially in East Africa. This study aimed to investigate the antimicrobial resistance profile and the genotypic relatedness of *Salmonella* Kentucky isolated from animal sources in Ethiopia and Kenya (n=19). We also investigated population evolutionary dynamics through phylogenetic and pangenome analyses with additional publicly available *Salmonella* Kentucky ST198 genomes (n=229). All the 19 sequenced *Salmonella* Kentucky isolates were identified as ST198. Among these isolates, the predominant genotypic antimicrobial resistance profile observed in ten (59.7%) isolates included the *aac(3)-Id*, *aadA7*, *strA*-*strB*, *bla*_TEM-1B_, *sul1*, and *tet*(A) genes, which mediated resistance to gentamicin, streptomycin/spectinomycin, streptomycin, ampicillin, sulfamethoxazole and tetracycline, respectively; and *gyr*A and *par*C mutations associated to ciprofloxacin resistance. Four isolates harbored plasmid types Incl1 and/or Col8282; two of them carried both plasmids. *Salmonella* Pathogenicity islands (SPI-1 to SPI-5) were highly conserved in the 19 sequenced *Salmonella* Kentucky isolates. Moreover, at least one Pathogenicity Island (SPI 1–4, SPI 9 or C63PI) was identified among the 229 public *Salmonella* Kentucky genomes. The phylogenetic analysis revealed that almost all *Salmonella* Kentucky ST198 isolates (17/19) stemmed from a single strain that has accumulated ciprofloxacin resistance-mediating mutations. A total of 8,104 different genes were identified in a heterogenic and still open *Salmonella* Kentucky ST198 pangenome. Considering the virulence factors and antimicrobial resistance genes detected in *Salmonella* Kentucky, the implications of this pathogen to public health and the epidemiological drivers for its dissemination must be investigated.

## Introduction

Salmonellosis accounts for approximately 25% of all diarrheic infectious diseases in humans, ranging from mild and self-limiting infections to fatal cases (WHO, 2018). Although more than 2,500 different *Salmonella enterica* serovars have been reported so far, most infections in humans are not caused by those that are highly adapted to humans, such as Typhi and Paratyphi, but rather by serovars capable of infecting a wide range of hosts, also known as non-typhoid *Salmonella* serovars, including *Salmonella* Kentucky (Issenhuth-Jeanjean et al., 2014).

The patterns of occurrence of non-typhoid *Salmonella* serovars among host species change over time and these dynamics depend on epidemiological and evolutionary aspects involved in host adaptation (Jajere, 2019). In the last few decades, there has been an increasing occurrence of *Salmonella* Kentucky in broilers and chicken products, as well as in pigs (Mahindroo et al., 2019).

Two sequence types (STs) of *Salmonella* Kentucky are known to play a role in public health: ST152, which is mainly reported in poultry and dairy flocks, and ST198, which is mainly associated with human infections in Africa, South Asia, Middle East and Europe (Park et al., 2020), as well as commonly reported in poultry and cattle in these regions (Haley et al., 2019). The burden is especially related to multidrug-resistant *Salmonella* Kentucky strains, as they can cause hard-to-treat infections (NARMS, 2017). *Salmonella* Kentucky strains that are resistant to quinolones, cephalosporins, carbapenems, and sulfonamides have been isolated from poultry flocks in Africa (Abd-Elghany et al., 2015). In Europe, there are reports of recurrent enteric diseases caused by *Salmonella* Kentucky in patients that had recently returned from African countries (Seiffert et al., 2014).

In the present study, we conducted a genomic characterization focusing on antimicrobial resistance determinants and SPIs investigation of *Salmonella* Kentucky isolated from livestock in East Africa in order to provide evolutionary and epidemiological insights into this serovar. We also investigated the evolutionary dynamics through phylogenetic and pangenome analyses with public genomes of 229 *Salmonella* Kentucky.

## Materials and Methods

### Bacterial Strains and Study Design

Nineteen isolates of *Salmonella* Kentucky originated from pig (12) and cattle (3) feces, and from chicken (2), pig (1) and cattle (1) tissues were isolated from Ethiopia and Kenya. Samplings were performed between 2005 (15 isolates) and 2013 (4 isolates). Briefly, samples were pre-enriched in buffered peptone water (37°C; 18 hours) and then enriched in both Rappaport Vassiliadis (1:100) and tetrationate broth (1:10) for 24 hours at 42°C and 37°C, respectively. Afterwards, samples were streaked onto brilliant green (BG) and XLT-4 agar dishes. Presumptive *Salmonella* spp. colonies per plate were tested biochemically by means of urea, triple sugar iron (TSI) and lysine iron (LIA) agar slants. Salmonella isolates were confirmed by slide agglutination test using poly-O antisera. Only one isolate from each sample was considered for whole genome sequencing in this study.

The isolates were deposited in the bacterial collection of the Infectious Disease and Molecular Epidemiology Laboratory (IDMEL), Ohio State University (OSU). Detailed information on *Salmonella* Kentucky isolates is shown in **Table 1**.

**Table 1:** *Salmonella enterica* serovar Kentucky ST198 isolates (n=19) cultured from animal sources in Ethiopia and Kenya.

Isolate ID	Accession Number^a^	Isolation date	Isolate Source	Host species	Origin	Antimicrobial Resistance Profile^c^	Clade
16584^b^	SRR2534093	11/20/2013	Feces	Swine	Nairobi, Kenya	SUT- CIP	B
11582^b^	SRR3027717	01/01/2005	Carcass	Swine	Addis Ababa, Ethiopia	AMP-CIP	A
11576	SRR3027712	01/01/2005	Chicken Carcass	Poultry	Addis Ababa, Ethiopia	AMP-STR-SUT-TET-GEN-CIP	A
11577	SRR3027719	01/01/2005	Beef	Bovine	Addis Ababa, Ethiopia	AMP-STR-SUT-TET-GEN-CIP	A
11578	SRR3027706	01/01/2005	Chicken Carcass	Poultry	Addis Ababa, Ethiopia	AMP-STR-SUT-TET-GEN-CIP	A
11579	SRR3027714	01/01/2005	Cecal contents	Swine	Addis Ababa, Ethiopia	AMP-STR-SUT-TET- CIP	A
11580	SRR3027721	01/01/2005	Cecal contents	Swine	Addis Ababa, Ethiopia	AMP-STR-SUT-TET-GEN-CIP	A
11581	SRR3027711	01/01/2005	Mesenteric lymph nodes	Swine	Addis Ababa, Ethiopia	AMP-STR-SUT-TET-GEN-CIP	A
11583	SRR3027707	01/01/2005	Mesenteric lymph nodes	Swine	Addis Ababa, Ethiopia	AMP-STR-SUT-TET-GEN-CIP	A
11584	SRR3027716	01/01/2005	Liver	Swine	Addis Ababa, Ethiopia	STR-SUT-TET-GEN	A
11589	SRR3027715	01/01/2005	Carcass	Swine	Addis Ababa, Ethiopia	STR-SUT-TET-GEN	D
16846	SRR3115978	12/27/2013	Feces	Bovine	Addis Ababa, Ethiopia	AMP-STR-SUT-TET-GEN-CIP	C
16845	SRR3115968	12/27/2013	Feces	Bovine	Addis Ababa, Ethiopia	AMP-STR-SUT-TET-GEN-CIP	C
16847	SRR3115987	12/27/2013	Feces	Bovine	Addis Ababa, Ethiopia	AMP-STR-SUT-TET-GEN-CIP	C
11586	SRR3027710	01/01/2005	Cecal contents	Swine	Addis Ababa, Ethiopia	STR-SUT-TET-GEN-CIP	A
11585	SRR3027723	01/01/2005	Tongue	Swine	Addis Ababa, Ethiopia	SUT-TET-GEN-CIP	A
11587	SRR3027720	01/01/2005	Liver	Swine	Addis Ababa, Ethiopia	STR-SUT-TET-GEN-CIP	A
11588	SRR3027708	01/01/2005	Mesenteric lymph nodes	Swine	Addis Ababa, Ethiopia	STR-SUT-TET-GEN-KAN-CIP	C
11590	SRR3027722	01/01/2005	Tongue	Swine	Addis Ababa, Ethiopia	STR-SUT-TET-GEN-CIP	C

a Accession number of sequences from GenBank;

b Isolates with no multidrug resistance profile.

c AMP, ampicillin (10 µg); CIP, ciprofloxacin (5 µg); GEN, gentamicin (10 µg); KAN, kanamycin (30 µg); STR, streptomycin (10 µg); SUT, sulfamethoxazole/trimethoprim (23.75/1.25 µg); TET, tetracycline (30 µg).

For comparative genomics and evolutionary analysis, we also used all public genomes of *Salmonella* Kentucky ST198 (n=229) that were available at Enterobase (Zhou et al., 2020) as of April 2020. These public isolates from cattle, swine, and poultry were collected across Africa, Asia and both North and South Americas. Accession numbers, metadata, as well as plasmid and SPI profiles, and genotypic antimicrobial resistance patterns of these isolates are presented in **Table S1**.

### Antimicrobial Susceptibility Testing

The phenotypic antimicrobial susceptibility of the 19 *Salmonella* Kentucky isolates was determined by means of the disk diffusion method, according to the documents of the Clinical and Laboratory Standards Institute (CLSI, 2018; CLSI, 2020). For that purpose, we used a panel of 12 antibiotics included in the World Health Organization (WHO) list of critically important and highly important antimicrobials (WHO, 2019): ampicillin 10 µg (AMP), amoxicillin/clavulanic acid 20/10 µg (AMC), ceftiofur 30 µg (CTF), ceftriaxone 30 µg (CRO), cephalothin 30 µg (CEF), chloramphenicol 30 µg (CHL), ciprofloxacin 5 µg (CIP), gentamicin 10 µg (GEN), kanamycin 30 µg (KAN), streptomycin 10 µg (STR), sulfamethoxazole/trimethoprim 23.75/1.25 µg (SUT) and tetracycline 30 µg (TET). *Escherichia coli* strain ATCC 25922 (CLSI, 2020) was used for quality control purposes. The CLSI document VET01S (CLSI, 2020) was used for the interpretation of CTF breakpoints, while the CLSI document M100-S28 (CLSI, 2018) was used for CIP breakpoints. Human-derived clinical breakpoints for Enterobacteriales listed in CLSI document VET01S (CLSI, 2020) were used for AMP, AMC, CRO, CEF, CHL, GEN, KAN, STR, SUT and TET.

### Whole Genome Sequencing

Genomic DNA of the strains was extracted using a commercial kit (QIAmp Fast DNA Tissue, Qiagen, USA). After extraction, DNA integrity was evaluated by means of electrophoresis in 1% agarose gel and quantified in a spectrophotometer (Colibri, Titertek-Berthold, Germany). The genomic libraries were prepared using Nextera XT V2 kit (2 x 250 bp) according to the manufacture’s guidelines and sequenced in a paired-end mode on a MiSeq platform (Illumina, USA).

### Downstream Bioinformatic Analyses

Raw reads in Fastq files were analyzed using FastQC version 0.11.7 (Andrews, 2010). Low-quality reads and adapters were removed using Trimmomatic-0.38 (Bolger et al., 2014). The genomes were assembled using SPades (Bankevich et al., 2012). The sequence type (ST) of the strains were determined by MLST 2.0 (Larsen et al., 2012) available at Center of Genomic Epidemiology (CGE).

The annotation of the 19 *Salmonella* Kentucky genomes was performed by Prokka software (Seemann, 2014), as well as the public genomes of *Salmonella* Kentucky (n=229) which have already annotated at Enterobase. The genetic structure and pangenome analysis of this group of *Salmonella* Kentucky isolates were evaluated with Roary (Page et al., 2015) using default parameters. The identification of *Salmonella* Pathogenicity Islands (SPIs) was performed using SPIFinder (Roer et al., 2016) and results were compared with the Virulence Factors Database (VFDB, 2020) using as threshold 95% of the identity and 60% of the length for each gene integrating the Island. *Salmonella enterica* serovar Typhimurium LT2 (access number AE006468) was used as a reference strain for BLAST Ring Image Generator (BRIG) (Alikhan et al., 2011).

Additionally, antimicrobial resistance determinants were investigated by means of ResFinder (Zankari et al., 2012) and plasmid profiling determined by PlasmidFinder (Carattoli et al., 2014), both performed with at least 95% of identity and 60% of minimum length, using CGE server. Morpheus online software (2020) was used to generate presence/absence maps, and hierarchical clustering was generated using “one minus Pearson correlation”.

### Phylogenetic Analysis

A phylogenetic analysis comprising 248 genome sequences of *Salmonella* Kentucky was performed by means of the maximum likelihood (ML) method, using RAxML v. 8.2.10 (Stamatakis, 2014) with default parameters. Clade support estimates were calculated using rapid bootstrapping of 1,000 pseudo replicates. GTR+G+I was applied as the best-of-fit model according to the Akaike Information Criterion (AIC) by means of jModeltest v. 2.1.10 (Posada, 2008). The phylogenetic tree was constructed using the core genome generated by Roary tool as input. The sequences derived from the core genome determined by Roary tool were codon aware aligned in PRANK (Löytynoja, 2014). *Salmonella* Typhimurium LT2 (accession: AE006468) was used as outgroup and to root the tree.

### Data Availability And Accession Numbers

The raw data of experimental isolates (n=19) are deposited at the NCBI Sequence Read Archive (SRA) web site, under Bioproject PRJNA275961. Accession numbers are provided in **Table 1**. Details on the 229 publicly available *Salmonella* Kentucky genomes from GenBank database are shown in **Table S1**.

## Results

### Antimicrobial Susceptibility Testing And Antimicrobial Resistance Determinants

All 19 sequenced *Salmonella* Kentucky isolates were identified as ST198. High resistance rates were observed for sulfamethoxazole/trimethoprim (18; 94.7%), ciprofloxacin (17; 89.5%), tetracycline (17; 89.5%), streptomycin (16; 84.2%), and gentamicin (16; 84.2%). None of the 19 *Salmonella* Kentucky showed resistance to amoxicillin/clavulanic acid, ceftriaxone, ceftiofur or chloramphenicol, while only a single isolate was resistant to kanamycin (5.2%). The antimicrobial resistance profiles of 19 *Salmonella* Kentucky isolates against the tested antimicrobial agents are shown in **Table 1**.

Among the eleven different resistance profiles, the most frequent one (AMP-STR-SUT-TET-GEN-CIP) was shared by nine (47.4%) isolates, while SUT-CIP and AMP-CIP, and STR-SUT-TET-GEN profiles were identified in one (5.2%) and two (10.52%) isolates, respectively. Seventeen (85%) isolates were resistant to three or more classes of antimicrobials and were considered multidrug-resistant (MDR) *Salmonella* Kentucky (**Table 1**).

All the 17 (89.5%) isolates showing phenotypic resistance to aminoglycosides harbored the genes *aac(3)-Id* [gentamycin (accession: AB114632)], while the gene *aadA7* [streptomycin/spectinomycin (accession: 161 AF2247)], and the genes *strA* [streptomycin (accession: AF321551)] + *strB* [streptomycin (accession: M96392)] were found in 16 (84.2%) and 13 isolates (68.4%), respectively. The genes *sul*1 [sulfamethoxazole (accession: U12338)] and *tet*(A) [tetracycline (accession: AJ517790)] encoding for resistance to sulfonamides and tetracyclines were found respectively in 18 (94.7%) and 17 (89.5%) isolates. *bla*_TEM-1B_ [ampicillin (accession: JF910132)] was the only ß-lactamase-encoding gene detected among the isolates (n=11; 57.9%). Accumulation of mutations in both GyrA (S83F/D87G) and ParC (T57S/S80I) conferring resistance to ciprofloxacin was found in 17 (89.5%) isolates, while two susceptible isolates (11589 and 11584) showed only single amino acid changes in both GyrA (S83F) and ParC (T57S). No plasmid-mediated quinolone resistance genes (PMQR) were identified among the 19 isolates. In terms of genotypic resistance, a total of six profiles were observed. Ten (52.6%) isolates presented the profile *aac(3)-Id*, *aadA*7, *strA*, *strB*, *bla*_TEM-1B_, *sul*1, *tet*(A), while four isolates (21.1%) had the profile *aac(3)-Id, aadA7*, *sul*1, *tet*(A).

Discordant genotypic and phenotypic resistance profiles were observed in isolates 11579 (harboring the *aac(3)-Id* gene) and 11585 (harboring the *aadA7* gene) that were susceptible to gentamycin and streptomycin, respectively. Moreover, neither aminoglycosides nor β-lactam resistance determinants were respectively found in the genome sequences of the phenotypically resistant isolates 11586 (AMP and STR) and 11588 (AMP and KAN).

**Figure 1:**
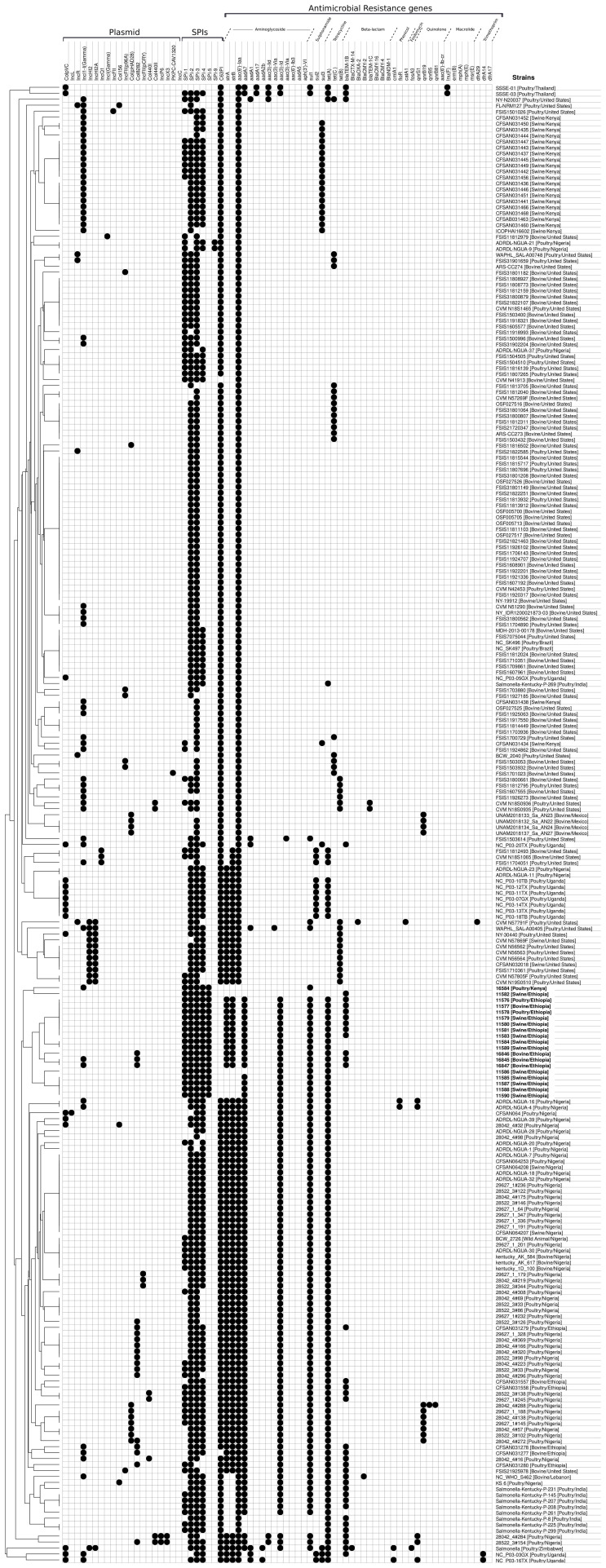
Antimicrobial resistance profile, pathogenicity islands and plasmid detected in both 19 *Salmonella* Kentucky isolates from the African Horn region and 229 publicly available *Salmonella* Kentucky genomes. The isolates were clustering according to the presence/absence of pathogenicity islands and plasmid, and antimicrobial resistance determinants as well.

The comparative genomic analysis including the other 229 *Salmonella* Kentucky ST198 genomes (**Table S1**) demonstrated the presence of the gene *aac(6’)-laa* in all of them. The genes *tet*(A) and *sul*1 were found in 44% and 28.4% of isolates, respectively, whereas *bla*_TEM-1B_ gene was detected in 14.8% of isolates (**Figure 1** and **Table S1**). A total of 99 (43.2%) genomes harbored antimicrobial resistance determinants against three or more drug classes and were considered multidrug-resistant genomes.

### Presence of SPI and Plasmids

**Figure 2:**
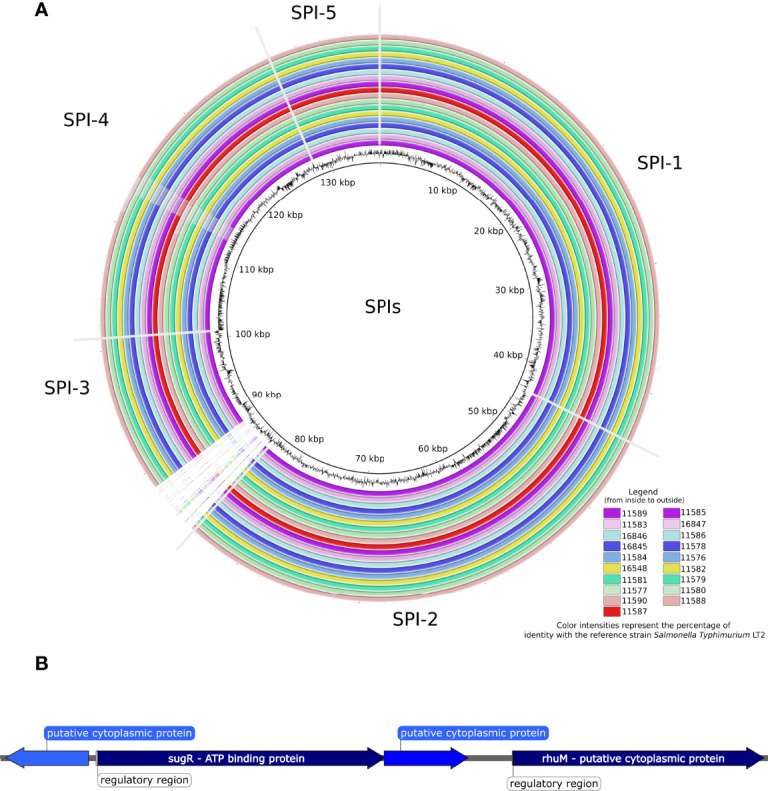
**(A)** BLAST ring image of the Pathogenicity islands detected in 19 *Salmonella* Kentucky isolates from the African Horn region. Color intensities represent the percentage of identity (> 90%) with the reference strain *Salmonella* Typhimurium LT2, while blank areas indicate no identity with the reference. The figure is shown in order from inside to outside, starting from the isolates in the left column. **(B)** The ~4kb-spanning region from SPI-3 that is absent in the 19 *Salmonella* Kentucky isolates.

We further evaluated the presence of *Salmonella* pathogenicity islands among the newly sequenced *Salmonella* Kentucky isolates (**Figures 1** and **2A**), as well as in the public 229 strains (**Figure 1** and **Table S1**). All 19 sequenced isolates carried five islands (SPIs 1, 2 and 5 showed 100% nucleotide identity, while SPIs 3 and 4 showed 82.7% and 92.5% nucleotide identity, respectively). Additionally, the 229 *Salmonella* Kentucky strains from the Enterobase database showed at least one pathogenicity island (SPI 1–5, SPI 9, or C63PI). The 19 isolates from Ethiopia and Kenya demonstrated high conservation of genes among SPIs (**Figure 2A**). The main differences were mainly found in the first 4kb of SPI-3, which harbors genes related to cytoplasmatic proteins (**Figure 2B**). However, the integration site related to the tRNA-selC appears to be conserved among all 19 *Salmonella* Kentucky isolates.

Plasmid amplicons were detected in the draft genomes of four experimental isolates (20.1%). Two different plasmid types were predicted: incompatibility group I1 [IncI1-Iγ – pR64 (accession: AP005147)] and colicinogenic factor [Col8282 - pMG828-3 (accession: DQ995353)]. Three isolates (15.8%) carried a single plasmid, while two isolates carried both plasmids IncI1-Iγ and Col8282 (**Table 2**). Among public isolates, we detected the Incl1-Iγ plasmid in 55 isolates (24%), while other plasmids, such as Col8282 (7.6%) and ColpVC (7.2%) were also detected at lower frequencies. One isolate from Nigeria was predicted to carry four distinct plasmids (IncX3, IncP6, Col440ll, and Col8282).

**Table 2:** Antimicrobial resistance determinants, mutations in GyrA and ParC (amino acid changes) and plasmids detected *in silico* in nineteen *Salmonella* Kentucky isolated from livestock in Ethiopia and Kenya.

Strain	Acquired Antimicrobial Resistance Genes^a^							Amino Acid Exchanges^b^		Plasmids	
	Aminoglycosides				β-lactams	Sulfonamide	Tetracyclines				
	*aac(3)-Id*	*aadA7*	*strA*	*strB*	*bla*_TEM-1B_	*sul1*	*tetA*	GyrA	ParC	Incl1-Iγ	*Col8282*
16584	–	–	–	–	–	+	–	S83F; D87G	T57S; S80I	+	–
11582	–	–	–	–	+	–	–	S83F; D87G	T57S; S80I	–	–
11576; 11577; 11578; 11579;11580; 11581 and 11583	+	+	+	+	+	+	+	S83F; D87G	T57S; S80I	–	–
11584 and 11589	+	+	+	+	–	+	+	S83F	T57S	–	–
16846	+	+	+	+	+	+	+	S83F; D87G	T57S; S80I	–	+
16845 and 16847	+	+	+	+	+	+	+	S83F; D87N	T57S; S80I	+	+
11586	+	–	–	–	–	+	+	S83F; D87G	T57S; S80I	–	–
11585; 11587; 11588 and 11590	+	+	–	–	–	+	+	S83F; D87G	T57S; S80I	–	–

a Acquired antimicrobial resistance genes mediating resistance to aminoglycosides [aac(3)-Id, aadA7, strA, strB], β-lactam [bla_TEM-1B_], sulfonamide [sul1] or tetracycline [tetA].

b Amino acid changes in GyrA and ParC. For the fluoroquinolones resistance-mediating mutations, please see the resistance to ciprofloxacin (CIP) in **Table 1**.

### Pangenome and Phylogenetic Analysis

**Figure 3:**
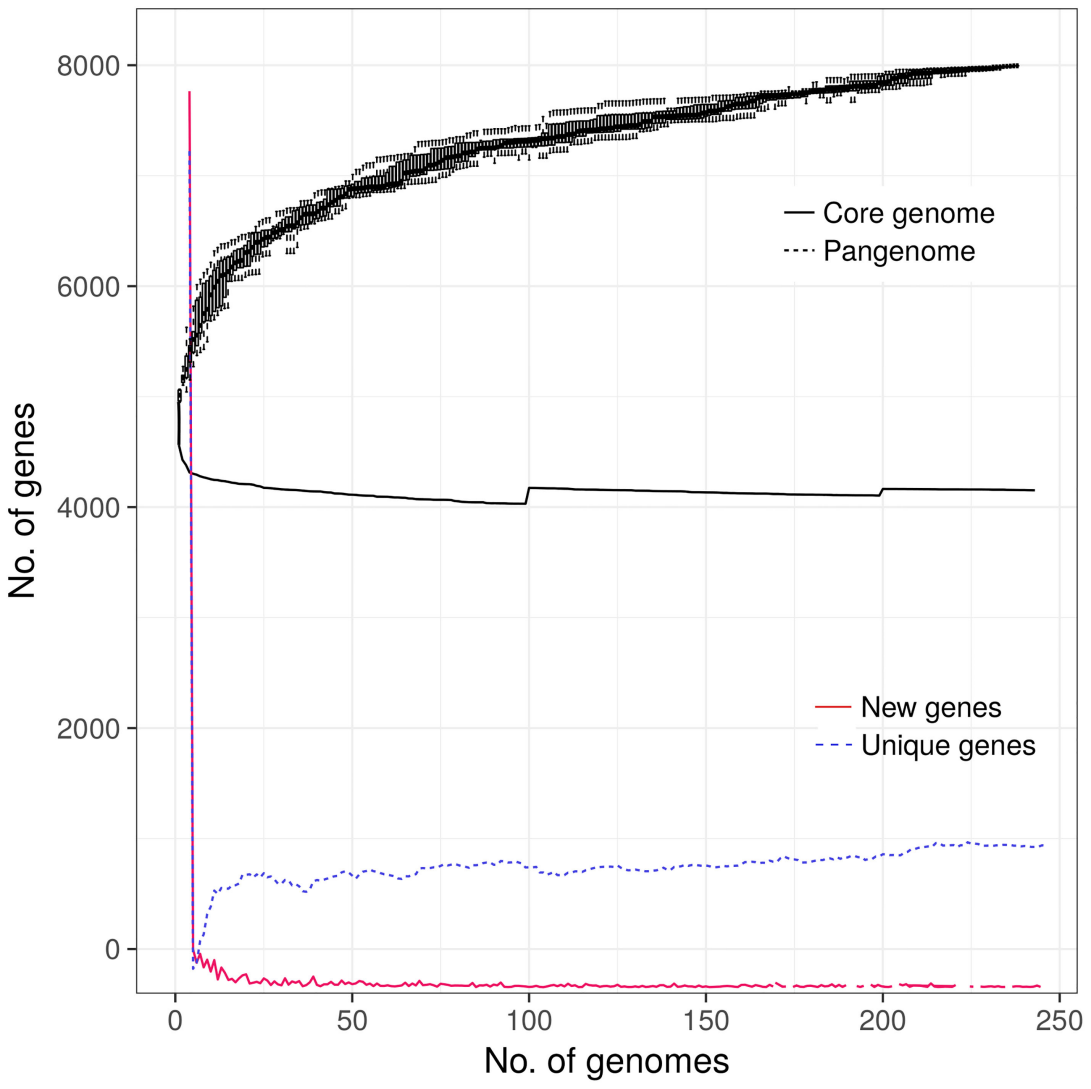
Pangenome analysis of 248 *Salmonella* Kentucky ST198 originated from farm animals. Including the genomes of 19 isolates originated from animal sources in the African Horn region (this study) and 229 additional genomes publicly available in the GenBank.

The pangenome analysis revealed that all 248 *Salmonella* Kentucky ST198 genomes harbored a total repertoire of 8,104 different genes (4,144 core genes, 122 soft core genes, 647 shell genes, and 3,191 cloud genes). The plateau zone observed in the curves for the core, unique and new genes (**Figure 3**), indicates that the core genome set has been already reached. However, the core and the accessory gene curves suggest that the pangenome of this group of isolates is still open (**Figure 3**).

**Figure 4:**
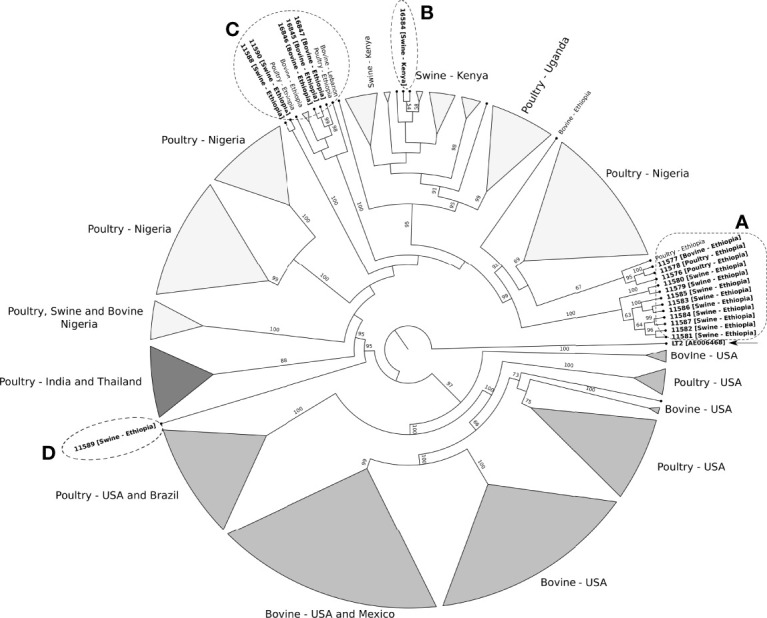
Maximum likelihood phylogenetic tree of 248 ST198 *Salmonella* Kentucky strains recovered from farm animals. *Salmonella* Typhimurium LT2 (accession number AE006468) was used as outgroup and to root the tree. The clades support is indicated above or next to each branch as bootstrap values, calculated from 1,000 pseudo replicates. Clades A to D include the nineteen isolates from Ethiopia and Kenya and other *Salmonella* Kentucky from the database. (**Figure S1** shown the detailed phylogenetic tree).

To further evaluate the phylogenetic relationships, we used the 4,144 core genes set of the 248 *Salmonella* Kentucky ST198. The clades generated by the phylogenetic tree were associated with the geographic source of the genomes (**Figure 4**). For instance, most swine isolates from Ethiopia formed a clade, closely related to poultry isolates from Nigeria (**Figure 4** - clade A). Additionally, isolate 16584 from pig feces in Kenya clustered together with other genomes from Kenya (**Figure 4** - clade B). Other swine and cattle isolates from Ethiopia were assigned to a new clade (**Figure 4** - clade C). Only one swine isolate (11589) from Ethiopia was closer to isolates from Asia and other African regions than to their Ethiopian counterparts. (**Figure 4** - clade D).

## Discussion

In view of the increasing public health threat posed by the global emergence and dissemination of antimicrobial resistant infectious agents, this investigation revealed a high level of both phenotypic and genotypic resistance to different classes of antimicrobials among 19 different strains of *Salmonella* Kentucky in Ethiopia and Kenya, as well as confirmed resistance to sulfonamides, tetracyclines, streptomycin, and gentamicin as common feature in *Salmonella* Kentucky serovars, corroborating previous reports (Abd-Elghany et al., 2015; Fernández et al., 2018; Neuert et al., 2018).

Some aspects make Africa a critical region in regard to the emergence of antimicrobial resistance (WHO, 2015), including deficient surveillance programs, production management failures, lack of adequate antimicrobial therapy, and proper regulation of antimicrobial use in food animals. The situation is particularly aggravated in this continent by the relatively large number of populations affected by Acquired Immunodeficiency Syndrome (AIDS), resulting in higher risks associated with co-infections by bacterial pathogens (Gordon et al., 2002).

Reports on the use of antimicrobials in food animals revealed that 14 drug classes have been largely used for non-therapeutic purposes in Africa, especially aminoglycosides, folate pathway inhibitors, ß-lactams, and quinolones, besides macrolides, which is not frequently used in livestock in non-African countries (Kimera et al., 2020). The frequencies of farms using antimicrobials vary between 77.6% in the Republic of Niger to 100% in Cameroon, Egypt, Ghana, Tanzania, and Zambia (Kimera et al., 2020). In Rwanda, the prophylactic use of antimicrobials was observed in about 97% of the farms; and 55.6% of farmers reported using these drugs without veterinary prescription or orientation (Manishimwe et al., 2017).

Overall, the prediction of antimicrobial resistance identified genes encoding for resistance to four different classes of antimicrobials in the 19 experimental isolates, which showed a high resistance rate to quinolones, folate pathway inhibitors, and aminoglycosides. Genes conferring resistance to aminoglycosides were detected in the majority of the *Salmonella* Kentucky genomes (17/19) and 12 of them harbored four aminoglycoside resistance determinants simultaneously [*aac(3)-Id*, *aadA*7, *strA*, and *strB*], corroborating Tyson et al. (2017). The disagreement between genotypic and phenotypic profiles for aminoglycoside resistance as observed in isolates 11579, 11585, 11586 and 11588 from our study has been previously observed in *Salmonella enterica* serovars (Cooper et al., 2020; Sia et al., 2021). The mechanisms associated with these incongruous findings should be further investigated. Regarding the isolate 11579, no difference has been found either in the genetic environment or gene sequence compared with other isolates harboring the same *aac(3)-Id* gene.The *aac(6’)-Iaa* gene was detected in all 229 genome sequences from the database. This gene may confer resistance to various aminoglycosides, such as amikacin, kanamycin and tobramycin, by means of the production of aminoglycoside-modifying enzymes (Salipante and Hall, 2003). Other genes encoding resistance to aminoglycosides (*aac(3)-Id*, *aadA*7, *strA* and *strB*) were commonly identified in the 19 sequenced isolates as well as in the 229 publicly available genomes. Our findings support previous report on the limited clinical use of aminoglycosides in infections caused by *Salmonella* (Fernández-Martínez et al., 2015). Resistance to sulfonamides and tetracyclines in the sequenced strains were mainly attributed to *sul*1 and *tet*(A), respectively. These genes have been frequently found in several *Salmonella enterica* serovars worldwide (Tyson et al., 2017; Neuert et al., 2018) and might be related to the to the extensive use of sulfonamides and tetracyclines in animal production systems (Mthembu et al., 2019). This is supported by the fact that *tet*A and *sul*1 were also the most prevalent resistance genes among the 229 *Salmonella* Kentucky sequences from the database. The gene *sul*1 has been highly frequent in *Salmonella* isolates recovered from retail food in Europe, with potential implications to public health (Maka et al., 2015).

A variety of genes conferring resistance to β-lactams are frequently found in *Salmonella enterica* (Neuert et al., 2018), including variants conferring resistance to third- and fourth-generation cephalosporins and carbapenems (Neuert et al., 2018; Fernández et al., 2018). Eleven out of the nineteen sequenced isolates in our study harbored the *bla*_TEM-1B_ gene, similarly to what we found in the other 229 public genome sequences of *Salmonella* Kentucky. These results support previous findings on the high frequency of *bla*_TEM-1B_ resistance gene in *Salmonella enterica* (Zhang et al., 2018). No β-lactam resistance genes were detected in two isolates (11586 and 11588) that were phenotypically resistant to ampicillin. Although not frequently observed, similar findings have been recently reported (Sia et al., 2021).

The resistance to quinolones in *Salmonella* Kentucky isolates observed in our study is in line with its occurrence reported in various *Salmonella* serovars worldwide (Tyson et al., 2017). In contrast to single mutations, the combination of multiple amino acid changes in GyrA (amino acids 83 and 87) and ParC (amino acids 57 and 80) has been reported to confer full ciprofloxacin resistance in *Salmonella enterica* (Eaves et al., 2004; Neuert et al., 2018). Moreover, all 19 isolates harbored the AcrAB-TolC efflux system related to antimicrobial resistance by substrate transport. Overexpression of this system due to mutations on regulation genes leads to MDR phenotype in Enterobacteriales (Grimsey et al., 2020). However, in our study, no mutation has been identified in *acrAB*, *tolC* or *ramA* genes.

No correlation between resistance genes and SPIs has been found. The antimicrobial resistance genes of the investigated isolates are clustered in a ~14Kb region, which includes genes conferring resistance against aminoglycosides, sulfonamide and tetracyclines, as well as the operon conferring resistance to mercur. However, the *bla*_TEM-1B_ gene has been found neither inside nor bordering this region, rather in different regions in the genomes that harbor it. Although the backbone of this 14Kb region resembles the *Salmonella* Genomic Island 1 variant (SGI1-K), typical genes as *xis*, *rep*, and orfs A and B have not been found (Doublet et al., 2008; Le Hello et al., 2011; Le Hello et al., 2012).

*Salmonella* Pathogenicity Islands (SPIs) were found in almost all investigated isolates. Homologs of SPIs 1, 2, 3, 4, 5 and 9 and the pathogenicity island of centisome 63 (C63PI) were detected in all 248 *Salmonella* Kentucky genomes. The presence of SPIs 1-5 highlights the pathogenicity of the sequenced strains, as these harbor several virulence genes that are crucial to trigger disease in the hosts (Hensel, 2004). SPI-1 harbors highly conserved genes among *Salmonella* serovars (Ramos-Morales, 2012), such as those responsible for the type III secretion system (T3SS) (Dhanani et al., 2015), which mediates the invasion in the host intestinal epithelium (Mirold et al., 2001). According to Hensel (2004), another T3SS is also encoded by genes present in SPI-2, facilitating the bacteria survival within the phagosome.

Missing regions in SPI-3 was the key difference between the *Salmonella* Kentucky isolates and the reference *Salmonella* Typhimurium LT2 strain. Deletion of genes in SPI-3 of different *Salmonella* serovars has been reported (Delgado-Suárez et al., 2018). All these deletions occurred in the 5’ region, the SPI-3 insertion site, a possible site for integrating new genes (Hensel, 2004).

In *Salmonella* serovars Typhimurium and Gallinarum, SPI-3 is located in the *sel*C tRNA locus with 17 kb size and approximately ten genes, such as *sug*R, *rhu*M, *rmb*A, *mis*L, *fid*L, *mar*T, *sls*A, *cig*R, *mgt*B and *mgt*C, that might play a role in virulence (Blanc-Potard et al., 1999; Rodrigues Alves et al., 2018). The genes from this region, as *mis*L, are involved in the intestinal colonization, including both adhesion and invasion of epithelial cells (Wang et al., 2018). On the other hand, the *mar*T gene has been associated to genomic and functional modifications in the SPI-3 region between different *Salmonella* serovars, favoring their adaptation to the hosts (Retamal et al., 2010).

SPI-3 also harbors the *mgt*CB operon, which encodes the MgtC virulence protein responsible for intramacrophage survival and the *mgt*B gene, a Mg^2+^ transporter (Retamal et al., 2009). Although deletions of *sug*R and *rhu*M genes in the 5’ region of SPI-3 has been reported in different *Salmonella* serovars (Zou et al., 2011), there is no previous investigation on this aspect in *Salmonella* Kentucky. In this context, the SPI-3 deletions observed in the 19 sequenced *Salmonella* Kentucky isolates in our study (**Figure 2**) may have occurred due to the constant evolution of the sequence distribution with insertions and deletions in the SPI-3 that may vary among serovars. However, gene deletions in these SPIs do not seem to influence the virulence of each *Salmonella* individually (Rychlik et al., 2009).

Conversely to SPI 3, we found conserved SPIs 4 and 5 in the sequenced *Salmonella* Kentucky. Considering the analysis of the 248 genomes of *Salmonella* Kentucky, SPI-4 was observed in 49.2% (122/248). This island represents a 27 kb length sequence which function is to encode the type I secretion system; however, little is known about its role on *Salmonella* virulence (Hensel, 2004). SPI-4 has been shown to be very conserved among several *S*. *enterica* serovars, except for the arrangement of six genes (Hensel, 2004). The loss of SPI-4 resulted in virulence attenuation of *Salmonella* Typhimurium and *Salmonella* Enteritidis serovars in mice (Kiss et al., 2007).

Plasmids are of major importance for the acquisition, maintenance, and transfer of antimicrobial resistance determinants (Dhanani et al., 2015). Their presence was investigated in this study, and IncI1-Iγ and Col8282 were found in four out of 19 isolates. IncI1-Iγ is characterized by a well-maintained dorsal skeleton, with a single site for integrating new genes (Johnson et al., 2011). Col type plasmids, on the other hand, have small deletions and/or insertions, except for the operon encoding colicins (Riley and Gordon, 1992), an important mechanism for competition among bacteria.

The IncI1-Iγ plasmid, found in 21.4% of the 248 genomes (**Table S1**), is frequently associated with the production of relevant β-lactamases, such as *bla*_CMY-2_, *bla*_CTX-M-1_, and *bla*_TEM-52_ (Smith et al., 2015). On the other hand, plasmids Col8282 and ColpVC were less frequent (**Table S1**). In a recent study, Col plasmids were shown to play a role in *Salmonella* Heidelberg survival in the poultry house environment (Oladeinde et al., 2018).

The phylogenetic data revealed that almost all strains of MDR *Salmonella* Kentucky ST198 stemmed from a single strain that has accumulated ciprofloxacin resistance-mediating mutations. This result may be strongly related to a high selective pressure generated by the overuse and misuse of fluoroquinolones in food animals, especially poultry, which are considered a major reservoir of *Salmonella* Kentucky strains (Shah et al., 2017). Kentucky ST198 serovar was reported to be susceptible to all antimicrobials until the 90’s (Weill et al., 2006). However, by means of acquisition of mobile elements carrying resistance genes, *Salmonella* Kentucky has become a widely spread multidrug-resistant serovar posing public health concerns in different regions (Hawkey et al., 2019). It was hypothesized that MDR *Salmonella* Kentucky strains stemmed from a single lineage that emerged in 1989 in Egypt before disseminating into Northern, Southern, and Western Africa, and then to the Middle East, Asia, and the European Union (Hawkey et al., 2019).

The fact that the pangenome of *Salmonella* Kentucky is still open suggests that the evolutionary dynamics of genetic variation in this serovar can lead to the emergence of MDR strains worldwide. Considering the increasing public health relevance of this serovar, with special emphasis to the MDR *Salmonella* Kentucky ST198 in livestock, our findings warrant further investigation to address the mechanisms associated with the emergence of this serovar.

## Conclusion

The potential public health burden associated with *Salmonella* Kentucky, especially in the African continent, is supported by the variety of antimicrobial resistance genes present in both mobile genetic elements and chromosomal DNA. The phenotypic and molecular mechanisms of antimicrobial resistance observed in our study support the hypothesis that the emergence and dissemination of multidrug-resistant *Salmonella* Kentucky might be associated with the overuse and misuse of antimicrobial agents in food animals, especially regarding (fluoro)quinolones, as the phylogenetic analysis revealed that almost all ciprofloxacin-resistant *Salmonella* Kentucky ST198 strains stemmed from a single strain that has accumulated ciprofloxacin resistance-mediating mutations. This information shed light on evolutionary and epidemiological aspects of *Salmonella* Kentucky in the scope of the increasing threat posed by the emergence and dissemination of antimicrobial resistance among zoonotic pathogens.

## Data Availability Statement

The datasets presented in this study can be found in online repositories. The names of the repository/repositories and accession number(s) can be found in the article/**Supplementary Material**.

## Author Contributions

MMSS, WAG, SK, and CJBO: Study conception and design. MMSS, NMVS, AMV, ADR, JAM, TE and VPB: Acquisition of data, analysis and interpretation of data. MMSS, VPB, AMV, ADR and OCFN: Drafting of the manuscript. WAG, ABJ and CJBO: Supervision. EJDS, JAM, TE, SK and CJBO: Critical revision. All authors read and approved the final manuscript.

## Funding

The authors wish to express their sincere gratitude for the financial support of Coordenação de Aperfeiçoamento de Pessoal de Nível Superior (CAPES, financial code 001; PDSE, 88881.131934/2016-01; Projeto CAPES-PrInt UFPB “Omic sciences applied to the prevention of antimicrobial resistance at the human-animal-environment interface-a one health approach” (88881.311776/2018-01/Bioma Caatinga, biodiversidade e sustentabilidade), Conselho Nacional de Pesquisa e Desenvolvimento (CNPq, proc 313678/2020-0), Sao Paulo Research Foundation (FAPESP, proc. 2018/21301-2), NIH Fogarty International Center (D43 TW008650), and FDA´s GenomeTrakr Network for the whole genome sequencing of isolates.

## Conflict of Interest

The authors declare that the research was conducted in the absence of any commercial or financial relationships that could be construed as a potential conflict of interest.

## Publisher’s Note

All claims expressed in this article are solely those of the authors and do not necessarily represent those of their affiliated organizations, or those of the publisher, the editors and the reviewers. Any product that may be evaluated in this article, or claim that may be made by its manufacturer, is not guaranteed or endorsed by the publisher.

## Supplementary Material

Supplementary Figure 1 Detailed Maximum likelihood phylogenetic tree of 248 ST198 *Salmonella* Kentucky strains recovered from farm animals. *Salmonella* Typhimurium LT2 (accession number AE006468) was used as outgroup and to root the tree.

Supplementary Table 1 Detailed information on all 248 *Salmonella* Kentucky from livestock in Africa, Asia, and America.
