# Supplementary figures and images for: Genomic and Evolutionary Analysis of Salmonella enterica Serovar Kentucky Sequence Type 198 Isolated From Livestock In East Africa
Source: Front Cell Infect Microbiol. 2022 Jun 20;12:772829. doi: 10.3389/fcimb.2022.772829 (PMC9251186; doi:10.3389/fcimb.2022.772829)

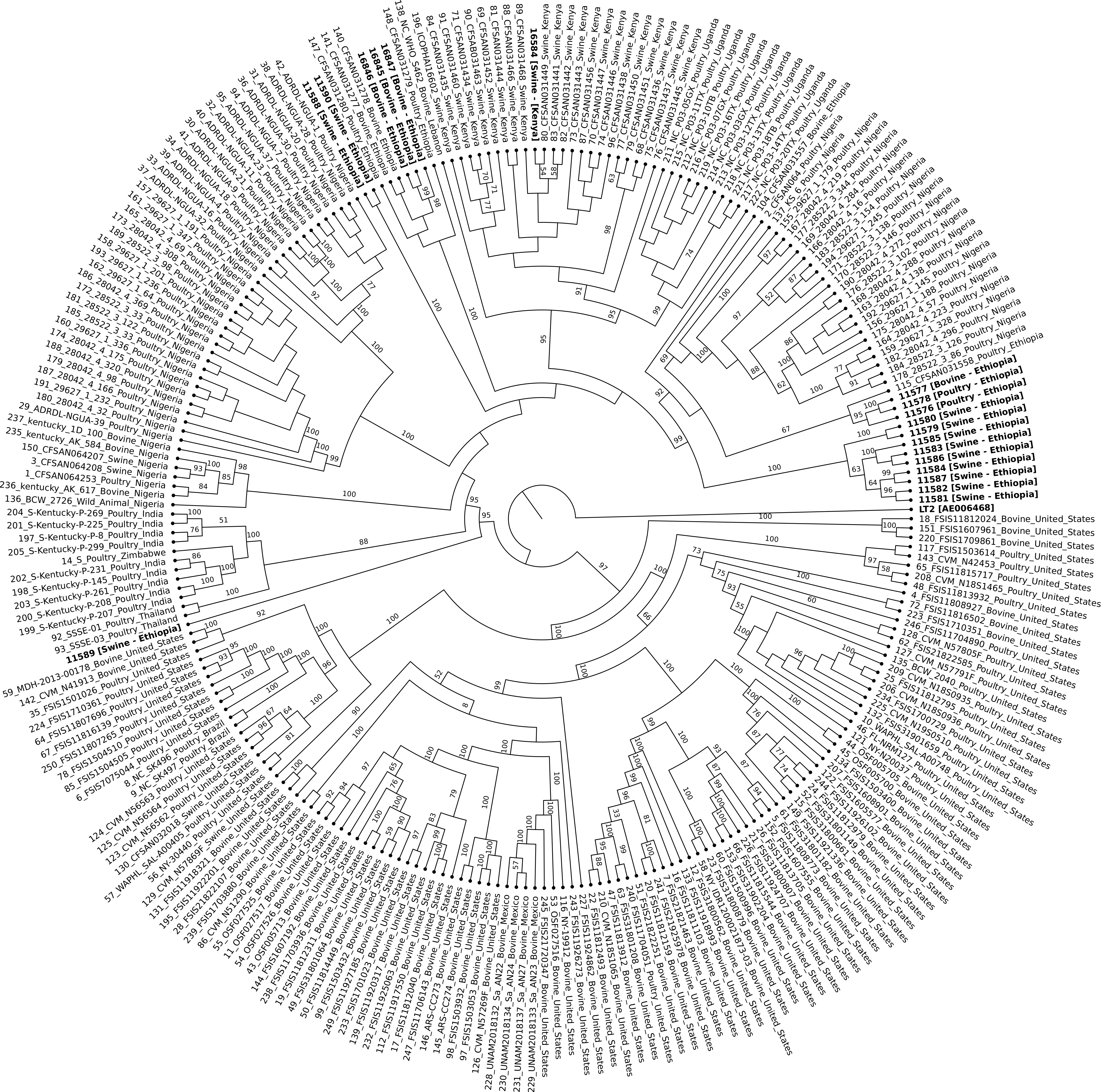

Supplement: Supplementary Figure 1 — Detailed Maximum likelihood phylogenetic tree of 248 ST198 Salmonella Kentucky strains recovered from farm animals. Salmonella Typhimurium LT2 (accession number AE006468) was used as outgroup and to root the tree. [file Image_1.tiff]
